# Self-induction system for cellulase production by cellobiose produced from glucose in *Rhizopus stolonifer*

**DOI:** 10.1038/s41598-017-10964-0

**Published:** 2017-08-31

**Authors:** Yingying Zhang, Bin Tang, Guocheng Du

**Affiliations:** 1 School of Biotechnology, Jiangnan University, Wuxi, 214122 China; 2 College of Biochemical Engineering, Anhui Polytechnic University, Wuhu, 241000 China; 3 Key Laboratory of Industrial Biotechnology, Ministry of Education, Jiangnan University, Wuxi, 214122 China

## Abstract

Cellulolytic fungi have evolved a sophisticated genetic regulatory network of cellulase synthesis to adapt to the natural environment. Even in the absence of lignocellulose, it still secretes low levels of “constitutive” cellulase for standby application. However, the mechanisms of this constitutive expression remain incompletely understood. Here we identified a cellobiose synthetase (CBS) from *Rhizopus stolonifer*, which has the capacity to catalyse the synthesis of cellobiose from uridine diphosphate glucose (UDPG). Through the construction of *R. stolonifer Δcbs* strain, we found that CBS plays a key role in the synthesis of cellulase. Further analysis of cellulase synthesis under glucose culture reveals that the cellobiose-responsive regulator CLR1 was activated by CBS-synthesized cellobiose, thereby promoting the expression of CLR2 and finally opening the transcription of cellulase genes. Our results suggest that *R. stolonifer* can be induced by self-synthesized cellobiose to produce cellulase, which can be used to reconstruct the expression regulation network to achieve rapid production of cellulase using simple carbon source. Based on our data, the “constitutive expression” of cellulase actually derives from the induction of cellobiose that synthesized by CBS from carbohydrate metabolites, which updates our knowledge of cellulase, and provides a novel insight into the regulation of cellulase synthesis.

## Introduction

Cellulase is generally divided into inducible and constitutive enzymes^1^. As previously reported, numerous induced hypotheses of cellulase that based on cellulosic substrates have been proposed^2–4^. The most recognized hypothesis is that low-level constitutive cellulase accomplishes the initial degradation of cellulose releasing the soluble oligosaccharides to induce the expression of cellulase^5–7^. However, there is no hypothesis about the genetics of cellulase constitutive expression, although the inducing abilities of various non-cellulosic substrates including glucose, lactose, and starch hydrolysate for cellulase production have been confirmed^8–10^. Furthermore, the acid-hydrolysed starch that mainly composed of glucose, dextrin, and other oligosaccharides, could be used as the main carbon source for efficient industrial production of cellulase from cellulolytic fungi. Therefore, confirming the synthesis mechanism of cellulase under the cultivation of non-cellulosic substrates is critical to subsequent regulation of production.

It has been reported that some disaccharides such as cellobiose, sophorose, and lactose have a strong ability to induce the expression of cellulase^11–14^. Moreover, sophorose has been found to be one of the converted products from cellobiose that trans glycosylated by β-glucosidase (BG)^15^. Cellobiose is recognized as a natural inducer for cellulase production^16^. A low-concentration of cellobiose can effectively induce the expression of cellulase in *Trichoderma reesei* ^9, 17^. Interestingly, we have detected a low-level cellobiose *in vivo* by growing *R. stolonifer* on glucose substrate (Supplementary Fig. 1). Therefore, we assume that there may be an enzyme which can synthesize cellobiose with glucose or its intermediate metabolites as precursors, which suggests that cellulase could be induced by the self-synthetic cellobiose.

Here we identified a glycosyltransferase CBS which may mainly account for catalysing the synthesis of cellobiose that participated in the induction of cellulase in *R. stolonifer*. To evaluate the role of CBS, we constructed *R. stolonifer Δcbs* and detected both transcription and expression levels of cellulase. The apparent differences between the parent and mutant strains suggested that CBS is one of the key proteins to control of cellulase synthesis. Based on the identification and characterization of CBS, we provided a new induction model which can describe the production pathway of cellulase using the non-cellulosic substrates.

## Results

### Identification of CBS from *R. stolonifer*

As reported in other cellulolytic fungi, cellobiose can effectively induce the secretion of cellulase in *R. stolonifer* (Supplementary Fig. 2). Our previous studies suggested that a cellobiose at low concentration level can be detected *in vivo* by growing *R. stolonifer* on glucose substrate (Supplementary Fig. 1a). Cellobiose production *in vivo* was then confirmed using liquid chromatography mass spectrometry (LCMS) comparing to a chemical standard (Supplementary Fig. 1b,c). Furthermore, the accumulation of cellobiose showed a correlation with the cellulase induction (Supplementary Fig. 1d). Since a low-level cellobiose was detected *in vivo*, there should be a system to synthesize cellobiose in *R. stolonifer*. Here, we chose cellulose synthase as a reference for the further study because of its capacity to catalyse the synthesis of cellulose *in vivo* by utilizing active glucose UDPG as glycosyl donors under glucose cultivation conditions. We hypothesized that the key catalytic structure of the “cellobiose synthase” should be similar to that of the cellulose synthetase, for which we compared the published cellulose synthase genes and designed the probe to capture the target gene from *R. stolonifer*. Fragment of the gene encoding conserved active centre of cellulose synthase (CESA domain) was used as a probe to extract target gene. The clone, *cbsh* (GenBank No. KT957546), resembles the fragment encoding CESA. Flanking sequences of *cbsh* were amplified by the TAIL-PCR. Finally, a novel gene, *cbs* (GenBank No. KT957545) was obtained and analysed by a multiple alignments of BLAST and ClustalW2. The results indicated that protein encoded by *cbs* belongs to glycosyltransferase with a partial characteristic sequence of BglB (Supplementary Fig. 3). The alignments of ClustalW2 showed that CBS contains an analogous CESA catalytic domain which was similar to the cellulose synthetase (GenBank No. XP_001390453) in *Aspergillus niger*. The percent identity calculated by MegAlign between CBS and this cellulose synthetase is 47.8%.

**Figure 1:**
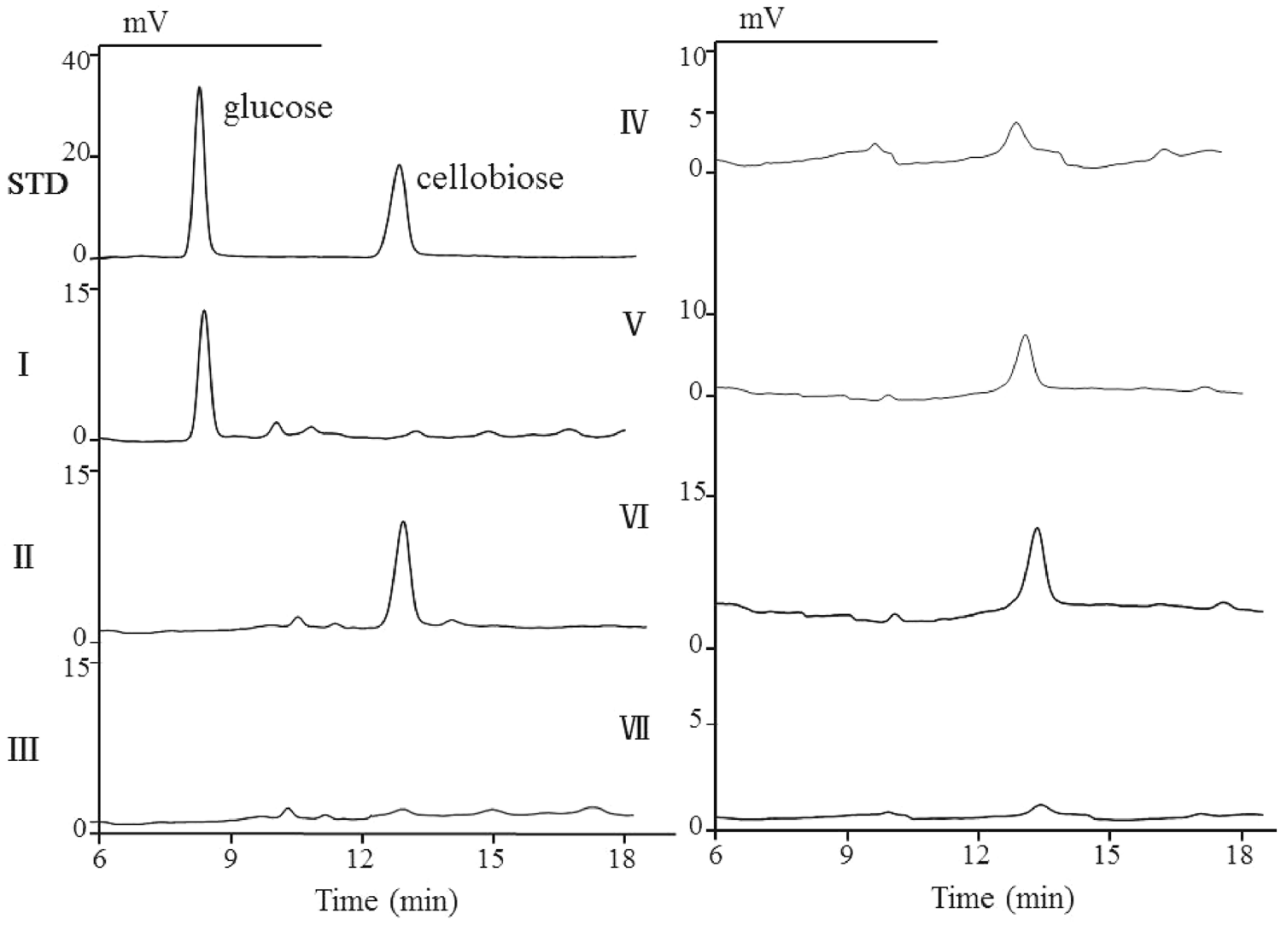
HPLC profiles of the fermentation extracts of *E. coli* BL21 (pET28a-*cbs*) and the components of CBS mixture. STD: 1% (m/V) glucose and 1% cellobiose; I: *E. coli* BL21 (pET28a); II: *E. coli* BL21 (pET28a-*cbs*); III: negative control of *E. coli* BL21 (pET28a-*cbs*) without induced; IV–VI: mixture of CBS (100 μg), UDPG (20 mg), and 0.5 mM ATP reacted at 30 °C for 30 min (IV), 1 h (V), and 1 day (VI); VII: negative control of CBS mixture without ATP.

**Figure 2:**
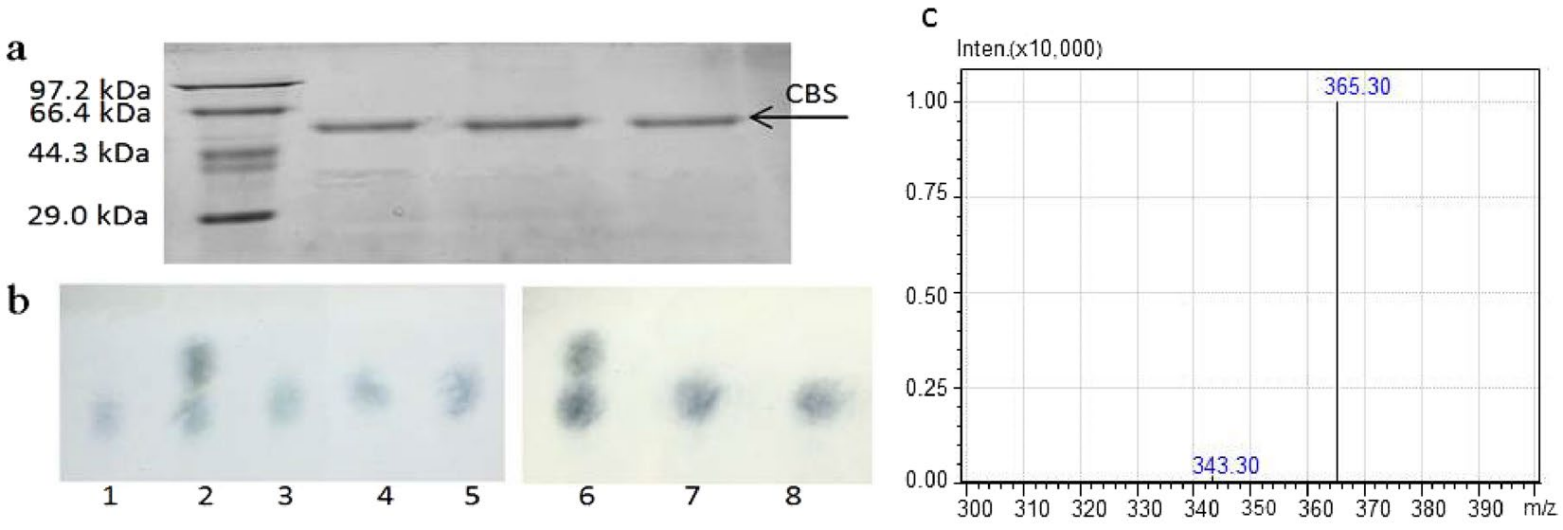
Characterization of CBS *in vitro*. (**a**) SDS-PAGE analysis of purified CBS (56 kDa). (**b**) Thin layer chromatography (TLC) results of the enzymatic reaction mixture of CBS. Line 1: 1% (m/V) cellobiose; Line 2, 6: 1% cellobiose and 1% sophorose; Line 3–5: mixture of CBS, UDPG, and ATP reacted at 30 °C for 1 day; Line 7: reaction mixture of cellobiose, ATP, and CBS; Line 8: negative control of cellobiose mixture with an inactivated CBS. (**c**) Liquid chromatography mass spectrometry (LCMS) results of the disaccharide synthesized by CBS.

To determine the function of CBS, the *cbs* gene within pET28a was expressed in *E. coli* BL21 (DE3). Positive clones were selected and cultured in a liquid LB medium with addition of glucose. Composition of the zymotic fluid was analysed by HPLC. The results indicated the presence of cellobiose, which suggested that the recombinant of *E. coli* was capable of synthesizing cellobiose (Fig. 1 and Supplementary Fig. 4). By the purification process using Nickel column, the target CBS with molecular weight of 56 kDa was obtained (Fig. 2a).

### Characterization of CBS *in vitro*

As one of the glycosyltransferases, CBS requires active nucleoside diphosphate sugars as glycosyl donors to catalyse the synthesis of cellobiose^18, 19^. So far, three nucleoside diphosphate glucoses including ADPG, UDPG and GDPG have been found^20^. The preferred glycosyl donor for synthesis of β-1,4-glucan in microorganism is UDPG^21, 22^. In order to identify the function of CBS, purified protein was tested *in vitro* at the simulating condition. The results showed that CBS has the ability to form a disaccharide by UDPG in the presence of ATP (Fig. 1 and Supplementary Fig. 4). However, this ability cannot be seen when the glucose was employed as substrate. This indicated that CBS may only act on the activated glucose in process needing energy. Then the enzymatic reaction mixture of CBS was analysed by thin layer chromatography (TLC) and LCMS. The results showed that the disaccharide synthesized by CBS *in vitro* is cellobiose (Fig. 2). Furthermore, cellobiose was used as the substrate to study whether CBS has the transglycosylation activity to turn cellobiose into sophorose. The results showed that CBS cannot transfer the cellobiose to form sophorose (Fig. 2b).

To examine the affinity of CBS with its substrates and product, we used Biacore T200 to study the interaction between those molecules. Affinity interaction was observed between CBS and three examined molecules including glucose. The equilibrium dissociation constant (K_D_) of CBS binding to UDPG, glucose or cellobiose was 2.09E-6 (M), 3.84E-6 (M) and 1.85E-5 (M), respectively (Supplementary Fig. 5 and S4 Table). The data indicated that CBS tends to affinity binding to UDPG and glucose even if the latter may represses the activity of CBS by competitive inhibition.

### Synthesis pathway of cellobiose *in vivo*

**Figure 3:**
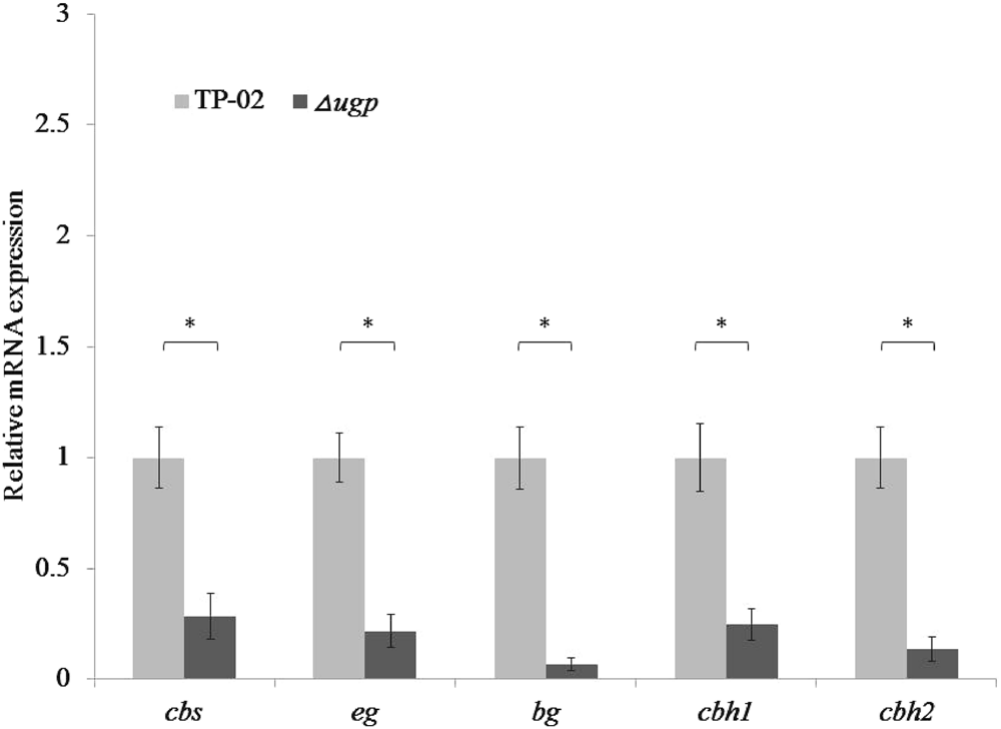
Comparison of wild-type *R. stolonifer* TP-02 and *Δugp* strains in the transcription level. The parent TP-02 and its *Δugp* mutant strain were cultured in a 2% glucose media. The deletion of *ugp* resulted in a sharply declined of normal transcription of *cbs* and cellulase genes. Error bars denote s.e.m. *P < 0.05.

UDPG, the preferred substrate for β-1, 4-glucan biosynthesis in both plant and bacteria^22–24^, is synthesized from glucose by Leloir pathway which involves the catalysis of glucokinase (GLK), phosphoglucomutase (PGM) and UDP-glucose pyrophosphorylase (UGP)^25–27^. In order to determine the synthesis pathway of cellobiose *in vivo*, we constructed *R. stolonifer Δugp* and researched the transcription level of related genes. Compared with the wild strain TP-02, the transcription level of *cbs* was sharply declined in the mutant strain. The results indicated that the deletion of *ugp* could influence the normal transcription of *cbs*. In particular, the transcription levels of four cellulase coding genes (GenBank No. JX315341, KF916015, KF916016, KP115896) were also sharply declined in *R. stolonifer Δugp* (Fig. 3). Furthermore, the sugar components *in vivo* of *R. stolonifer Δugp* cultured on glucose medium was analysed by HPLC. The results showed that texted mutants cannot synthesize cellobiose (Supplementary Fig. 6a). These results suggested that UDPG might be the major glycosyl donor for CBS to synthesize cellobiose. It could be due to the deficiency of *ugp* which hinders normal formation of UDPG, consequently, affects the synthesis of cellobiose and the transcription of the cellulase genes.

### Effect of CBS on the synthesis of cellulose

**Figure 4:**
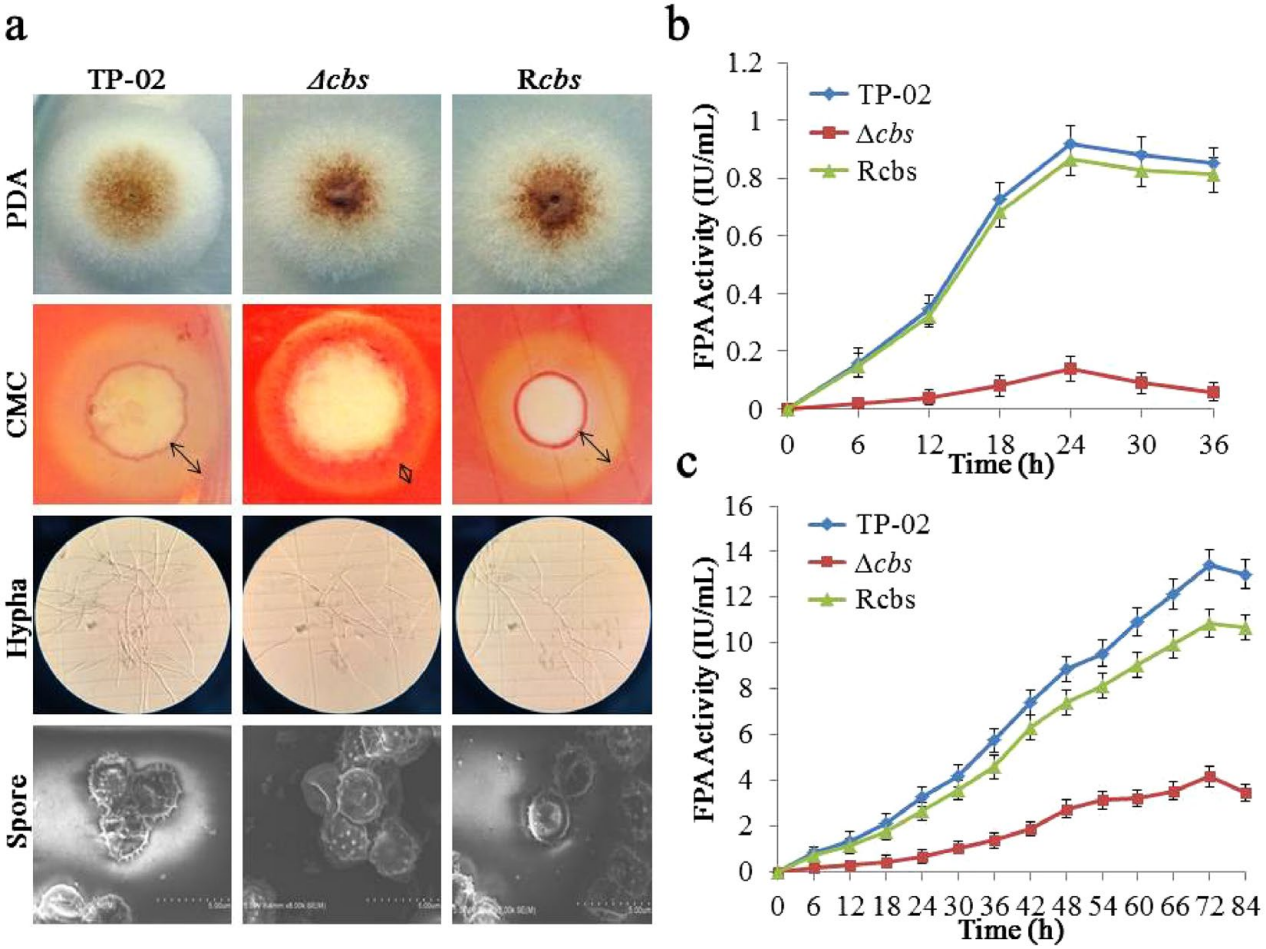
Phenotypic observations and cellulase production of *R. stolonifer* TP-02, R*cbs* and *Δcbs* strains. (**a**) The colony morphology (cultivated in PDA plates), Congo red staining (cultivated in 2% CMC plates), hypha shape and spore morphology (electron microscope) of TP-02, *Rcbs* and *Δcbs* were compared. The double-headed arrow marks the transparent circle. (**b**) Determination of the FPA activity of those three strains using 2% glucose (**b**) or 2% rice straw (**c**) as the single carbon source. The data are presented as mean ± s.e.m. from three independent experiments. The statistical significance was calculated with the t-test, *P < 0.05.

**Figure 5:**
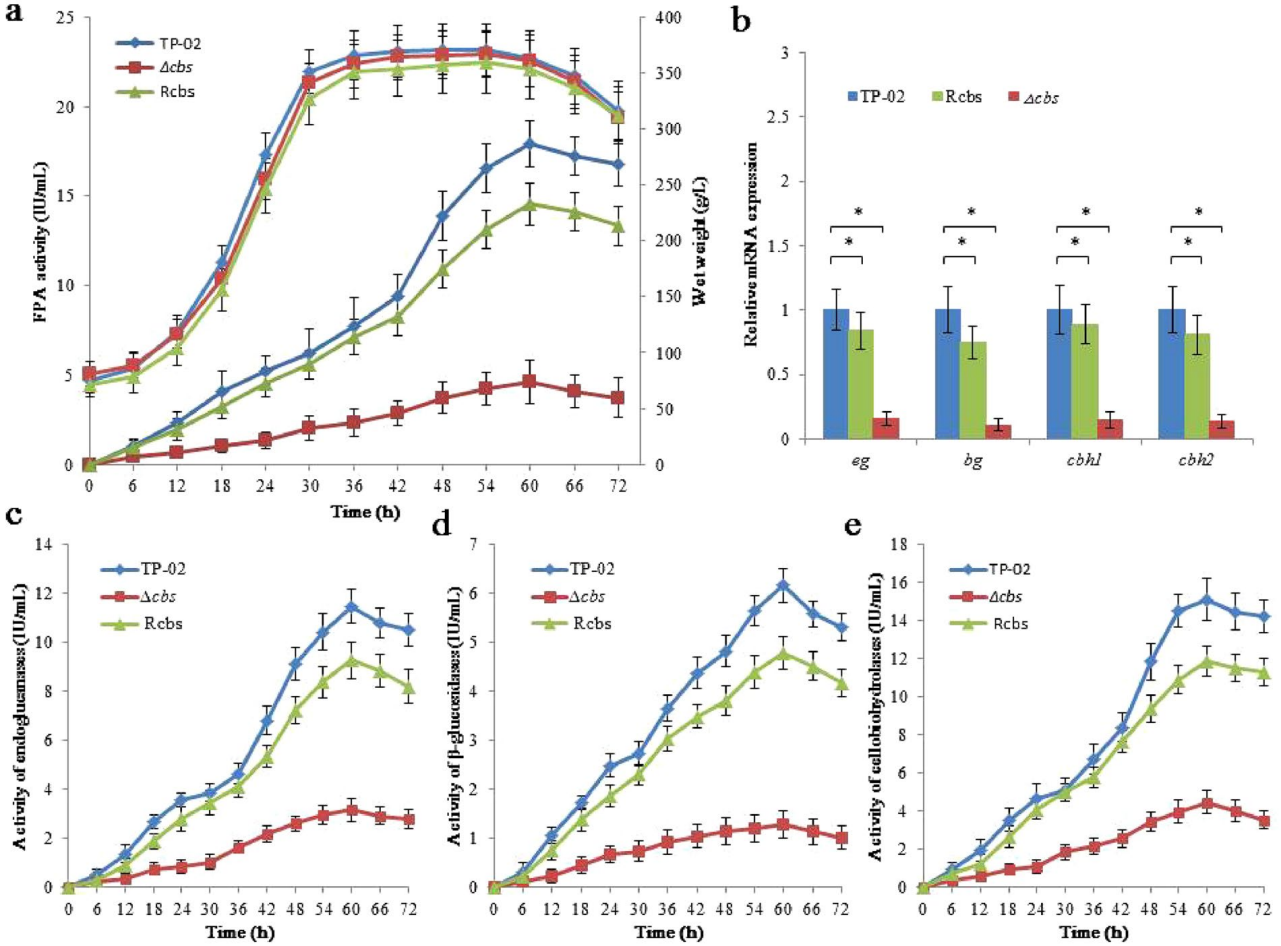
Comparison of *R. stolonifer* TP-02, R*cbs* and *Δcbs* strains from both the level of expression and transcription. (**a**) The wet weight and FPA activity of TP-02, *Rcbs* and *Δcbs* cultured in the fermentation medium contains10% starch hydrolyzate in 5 L fermenters. (**b**) Relative mRNA expression of cellulase genes from those three strains detected by RT-qPCR. (**c**) The activities of endoglucanase (**c**), β-glucosidase (**d**) and cellobiohydrolase (**e**) of those three strains. The data are presented as mean ± s.e.m. from three independent experiments. The statistical significance was calculated with the t-test, *P < 0.05.

To evaluate the effect of CBS on the synthesis of cellulase, we constructed the *R. stolonifer Δcbs* by homologous recombination using *R. stolonifer* TP-02 as the parent strain. Furthermore, we constructed the complementation strain R*cbs* to identify that the characteristics observed form *Δcbs* mutants were indeed caused by the deletion of *cbs*. Colony morphology and conidiation of those three strains were analysed. As shown in Fig. 4, no apparent changes of phenotypic were observed, suggesting that deficiency of *cbs* doesn’t substantially influence the spores and hyphae development in *R. stolonifer*. The results of Congo red staining showed that the deletion of *cbs* caused a decrease in CMC activity. The *R. stolonifer* parent, R*cbs* and *Δcbs* strains were inoculated on the starch fermentation medium in 5 L fermenters and cultured under the same conditions. The culture supernatants were analysed by SDS-PAGE (Supplementary Fig. 7) and cellulase assayed (Fig. 5). The activity of cellulase produced by *R. stolonifer Δcbs* (FPA activity is 4.61 IU/mL) is significantly lower than the parent strain (FPA activity is 17.93 IU/mL) and the R*cbs* strain (FPA activity is 14.54 IU/mL). Like the FPA activity displayed, the activities of endoglucanases, cellobiohydrolases and β-glucosidases produced by *R. stolonifer Δcbs* were also much lower than the parent (Fig. 5c–e). These results indicate that deficiency of *cbs* results in a reduced production of cellulase in *R. stolonifer*.

To show the effect of CBS, rice straw and glucose were used as single carbon source to culture *R. stolonifer* parent, R*cbs* and *Δcbs* strains. The results of cellulase production experiments indicated that deficiency of *cbs* could result in a reduced production of cellulase in both the cellulosic and non-cellulosic substrates (Fig. 4b,c). The activity of cellulase produced by *R. stolonifer Δcbs* (FPA activity is 4.15 IU/mL) is significantly lower than the parent strain (FPA activity is 13.42 IU/mL) and the Rcbs strain (FPA activity is 10.87 IU/mL) when induced by rice straw. Similarly, the FPA activities of *R. stolonifer* and its deficient strain *Δcbs* were 0.96 IU/mL and 0.14 IU/mL, respectively, when the glucose was used as carbon source.

We further analysed the transcription of cellulase coding genes in *R. stolonifer* parent, R*cbs* and *Δcbs* strains induced by starch hydrolyzate to confirm whether CBS controls the synthesis of cellulase. The transcription levels of these genes in those three strains were analysed by RT-qPCR and the results showed that they are significantly downregulated in *R. stolonifer Δcbs* (Fig. 5b).

### Induction pathway of cellulose

**Figure 6:**
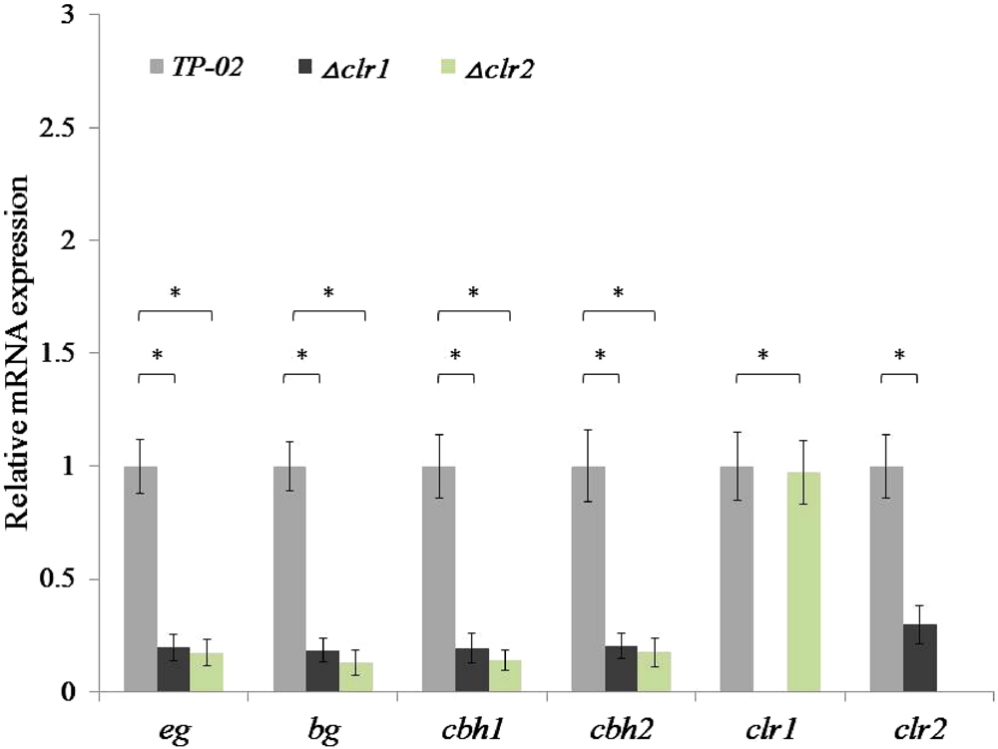
Comparative analysis of the transcription of cellulase genes from *R. stolonifer* TP-02 and its mutant strains. Data represent mean ± s.e.m. of triplicate experiments.*P < 0.05 by two-tailed t-test.

Cellobiose, which activates the transcription factor CLR-1 and promotes the expression of cellodextrin transporters, β-glucosidases and CLR-2, is required for the cellulase induction mediated by CLR-1 and CLR-2 in *Neurospora crassa* ^2, 28^. The cellobiose response regulator ClbR (CLR-2 homologous protein) of *Aspergillus aculeatus* has been confirmed to drive the expression of carbohydrate-active enzyme (CAZy) genes^29^. To study the induction pathway of cellulase regulated by CBS, we constructed the *R. stolonifer Δclr1*, and *Δclr2*. Through comparison the transcription levels of cellulase genes from parent and mutant strains cultured in glucose media, we determined that deficiency of *clr1* or *clr2* could result in a significant down-regulation of cellulase expression (Fig. 6). It indicates that the induction pathway controlled by cellobiose response regulator CLR is the main way to synthesize cellulase under the condition of glucose. Moreover, *clr1* is the key regulator of *clr2*, while the level of *clr2* plays a key role in this induction pathway of cellulase.

**Figure 7:**
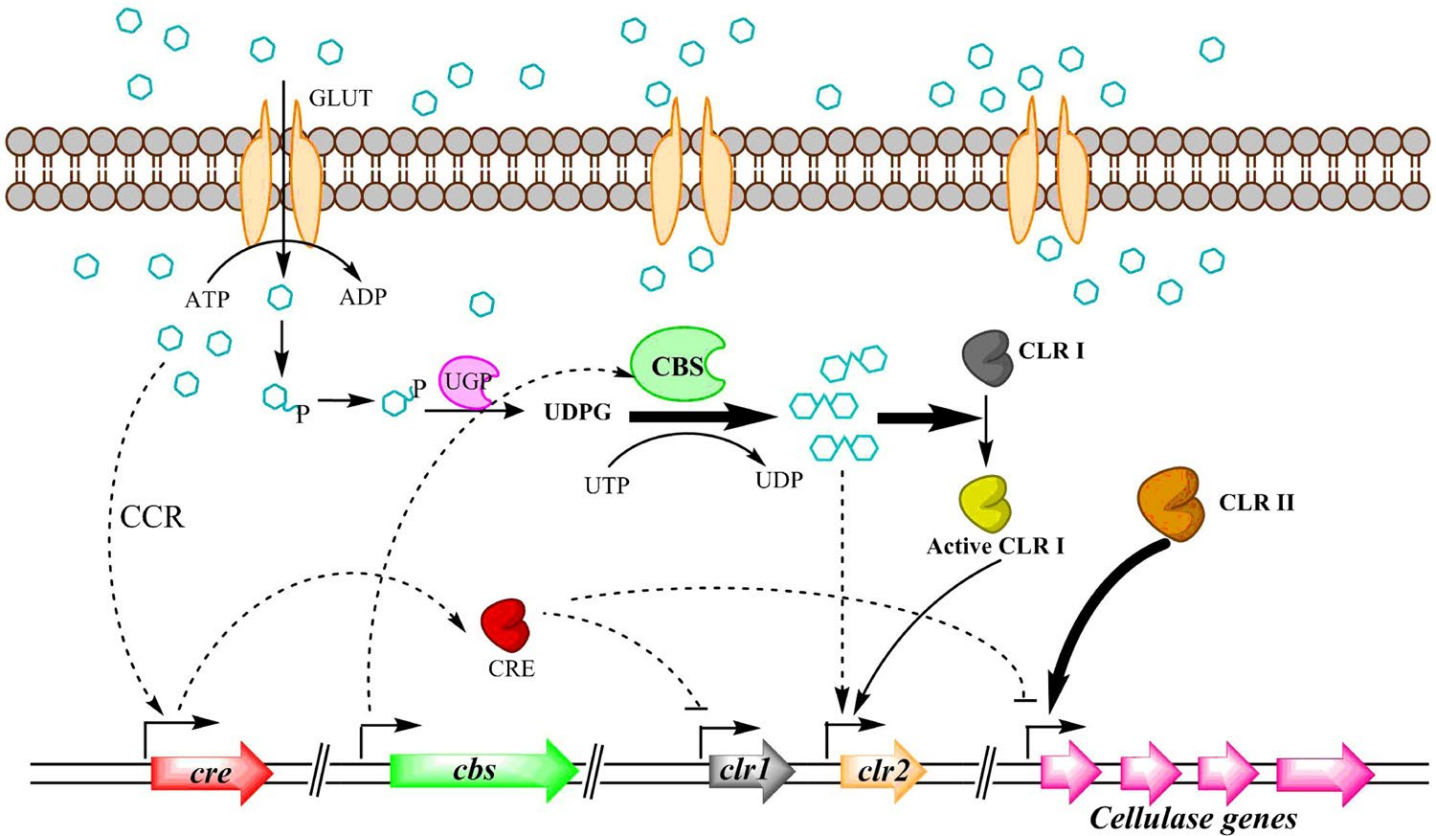
Hypothetical induction model of cellulase from *R. stolonifer* TP-02 under the cultivation of glucose. Glucose enters the cell through transporters GLUT and is converted to glucose-6-phosphate and glucose-1-phosphate step by step. The glucose-1-phosphate is catalysed by pyrophosphorylase (UGP) to form UDPG. Then cellobiose is synthesized by CBS using UDPG as the glycosyl donor. Through activating the cellobiose response regulator CLR1 and promoting the expression of CLR2, the self-synthetic cellobiose turns on the induction expression of cellulase. CCR refers to the carbon metabolic repressive effect that is a crucial regulation in microorganism preventing the expression of enzymes required for utilization of complex carbon sources when simple carbon sources like glucose are present in the medium.

Based on the above results, we designed a new induction model of cellulase (Fig. 7). Firstly, glucose was formed to the UDPG by a series of metabolic processes that catalysed by GLK, PGM, and UGP. Secondly, UDPG was catalysed by CBS to synthesize the cellobiose for opening the induction of cellulase. Finally, this self-synthetic cellobiose activated the CLR1 to promote the expression of CLR2, and then turn on the transcription of cellulase genes.

## Discussion

Previous studies often divide cellulase into inducible and constitutive two systems, which constrain the further regulation and modification of the entire cellulase system. Through the discovery and in-depth study of CBS from *R. stolonifer*, we determine that a cellulase self-inducing system has naturally evolved in *R. stolonifer*. By examining the purified CBS *in vitro*, it has been confirmed that CBS can synthesize cellobiose from UDPG that is the activated product of glucose catalysed by a series of enzymes in glycolysis^30^. Because UDPG can be developed from both cellulosic and non-cellulosic substrates by enzymolysis or thermal processing, we anticipate that the CBS would be the key enzyme participates in the control of cellulase synthesis. Its ability to catalyse cellobiose synthesis has an important role in explaining the induction synthesis of cellulase.

Although several models based on cellulosic substrates have been proposed, the non-cellulosic substrates containing glucose, oligosaccharide and starch sugar usually attract the attention on the carbon metabolic repression they caused, thereby ignoring the low expression levels of cellulase under these conditions^31^. This low level of expression might be derived from the induction of cellobiose generated by CBS. Furthermore, the concentration of UDPG *in vivo* should be in equilibrium, and accumulation of a large number of cellobiose could never be formed *in vivo*. It may explain the low levels of initial source of cellulase with cellulosic substrates, and provide a new view of the induction mechanism of cellulase synthesis. Based on the data, we construct a cellulase synthesis model of *R. stolonifer* under glucose condition, thus updating the cognition of “constitutive expression” theory of cellulase and providing a comprehensive model basis for subsequent genetic modification.

Because fermentation time is generally long and the production costs are high due to the characteristics of strains and the factors of culture conditions, filamentous fungi currently used in cellulase production is hardly to be fast and efficient^32^. The *R. stolonifer* has the capacity to secrete various cellulases and hemicellulases with faster enzyme production than *Trichoderma reesei*, showing an attractive industrial development and application potential. Based on successfully constructing a specific strain by regulation of enzymes participated in the UDPG synthetic pathway, and analysing the structure and function of CBS to engineer new protein molecules, our study provides a possible solution to achieve large-scale synthesis of cellulase since simple carbon source can be utilized to activate the synthesis of cellulase with a rapid development of cells.

## Materials and Methods

### Strains and reagents

*R. stolonifer* TP-02 was isolated in our laboratory and stored in China General Microbiological Culture Collection Center (CGMCC No. 11119). Sophorose, ATP, and UDPG were purchased from Sigma-Aldrich.

### Liquid chromatography mass spectrometry

An LC/MS/MS method was developed for the measurement of cellobiose produced by *R. stolonifer* TP-02 from glucose *in vivo* using a Shimadzu LCMS-8030 liquid chromatography mass spectrometer. The LC column used was an XBridge Amide (Waters). Mobile phase was 82% acetonitrile and 18% water. Measurement conditions: ESI; DL 250 °C; nebulizing gas 3 L/min; heat block 400 °C; drying gas 15 L/min.

### Identification of cellobiose synthetase encoding gene

The *R. stolonifer* TP-02 was cultured in PDA liquid medium and shaken for 24 h at 30 °C (200 rpm). The mycelium was harvested and freeze-dried. The genomic DNA was extracted by the standard CTAB method^33^. Amplification of a DNA fragment encoding a portion of CBS was done by the primers P1: 5′-CCKCRGGTSGACRTNNTG-3′ and P2: 5′-AWGCBGTYGAGAGTGGCC-3′. The PCR product was purified by the kit and sequenced by Sangon Biotech Co., Ltd. (Shanghai, China). The flanking sequences were amplified by TAIL-PCR^34^ with the primers listed in the Table S1. Homology alignment of the primary structure between CBS and other enzymes was carried out in the GenBank database using the BLAST program, together with the MegAlign program and the ClustalW2 (http://www.ebi.ac.uk/Tools/msa/clustalw2/).

### Protein expression and purification

The target gene was connected with pET28a vector and transformed into *E. coli* BL21 (DE3). The recombinants were cultured in LB liquid medium and shaken for 10 h at 37 °C (220 rpm), followed by switching to an improved TB medium (peptone 12 g/L, yeast extract 24 g/L, KH_2_PO_4_ 2.31 g/L, K_2_HPO_4_ 9.85 g/L, glycerol 9.85 g/L, pH7.0) with 1% inoculums size. Protein synthesis was induced by addition of 1 mM isopropyl β-D-1-thiogalactopyranoside at *OD*_600_ ~ 0.6. After protein expression for 18 h at 16 °C, cells were collected by centrifugation, re-suspended in PBS buffer and lysed by sonication. The cell lysate was cleared by centrifugation at 20,000 g for 45 min at 4 °C. The supernatant was loaded onto a His Trap FF column (GE Healthcare Life Sciences) attached to a ÄKTA Pure FPLC system (GE Healthcare) and washed with ten column volumes of wash buffer consisting of 20 mM Tris-HCl pH 7.5, 0.5 mM NaCl, 20 mM imidazole. The sample was eluted with the same buffer containing 400 mM imidazole. Samples were collected for SDS-PAGE electrophoresis verification.

### *In vitro* reactions

All reactions were performed in PBS buffer (pH 6.0, 50 mM). A mixture of the purified CBS (100 μg), 0.5 mM ATP and glucose (20 mg) or UDPG (20 mg) was suspended in 1 mL of PBS buffer and the mixture was stirring at 30 °C for 1 day. After reaction, the mixture was centrifuged and treated with filter membrane (pore size 0.45 μm). HPLC was utilized to analyze the composition of the samples. Measurement conditions: column, XBridge Amide (Waters); column temperature, 30 °C; mobile phase, 82% acetonitrile, and 18% water; flow rate, 0.4 mL/min; injection volume, 20 μL. Standard configuration: standard mixture was prepared by 1% mobile phase.

### Thin layer chromatography (TLC)

The reaction mixture of CBS, UDPG and ATP was further analysed by TLC. The stationary phase was 6 g silica gel GF254 dissolved by 0.02 M sodium acetate trihydrate (containing 0.5% sodium carboxymethyl cellulose). The developing solvent was n-butyl alcohol: ddH_2_O: acetic acid: ammonia water in 10: 5: 5: 1 (v/v). Colour developing agent was composed of two reagents: the first one is the mixture of aniline, diphenylamine and phosphoric acid which was prepared by dissolving 4 g aniline in 4 mL diphenylamine and adding 200 mL acetone and 20 mL 85% phosphoric acid; the second one is the mixture of 5% concentrated sulphuric acid dissolved by ethyl alcohol. These two mixed reagents were sprayed in the thin-layer plate respectively. Spots of carbohydrate components could be observed after heating at 105 °C for 5-10 min. The concentration of the entire master standard is 1% (m/v).

### Biacore analysis

Tests for adherence between CBS and micromolecules were performed in triplicate using a biosensor Biacore T200 recording SPR. CM7 (GE Healthcare) sensor chips were used to fasten CBS (300 µg/mL) in 14000 RU via amino coupling reaction. Binding experiments were carried out in the binding buffer of 50 mM NaAc, pH 4.5, 100 µM EDTA, at a flow rate of 30 µL/min. UDPG, glucose, cellobiose and maltotriose (negative control) were dissolved in gradient (0, 0.78, 1.56, 3.125, 6.25, 12.5, 25 µM) by 2 mM PBS buffer.

### Construction of *R. stolonifer* mutant strains

The full-length hygromycin resistance gene *hyg* was respectively inserted into *cbs*, *ugp*, *clr1*, and *clr2* by the overlapping PCR^35^. All primers used in the study were presented in the Table S2. The constructed genes were connected with the pUCm-T carrier and transformed into *E. coli* DH5α. The positive clones were screened and identified by sequencing the plasmid extracted by a kit. The pUCmT-*hyg*-*cbs*, pUCmT-*hyg*-*ugp*, pUCmT-*hyg*-*clr1*, and pUCmT-*hyg*-*clr2* were added to the pre-treatment spore suspension of *R. stolonifer* (germinated for 8 h), and placed on ice for 10 min before shifted into the pole cup. After shocking at 1500 V, the solution was immediately added to 1 mL pre-cooled PDA on ice for 20 min, and then cultured at 30 °C, 100 rpm for 90 min. Then, it was coated to resistant plates (hygromycin 180 μg/mL) and incubated at 30 °C. Preparation of the germinated spore: rinsed the spores with sterile water, filtrated by two layers of gauze, inoculated into PDA liquid medium, cultured at 30 °C, 180 rpm for 8 h until spore germination (electron microscopy was used to determine the spore germination time).

### Complementation experiments

The complementation cassette was used to transform into the *Δcbs* strains to identify that the interesting phenotypes observed for the mutants were indeed caused by the deletion of *cbs*. The *cbs* wild-type allele complementation cassette was amplified through the primer pairs *cbs*-F + *cbs*-R (Table S2). The pUCm-T-*cbs* was constructed and transformed into the *Δcbs* mutant. A PDA plate without hygromycin resistance was used to preliminary screening by incubating the transformant at 30 °C. The resistant plates (hygromycin 180 μg/mL) were used to secondary screening. The complementation strain R*cbs* was obtained and verified by amplifying *cbs* from the genomic DNA.

### Enzyme characterization of cellulose

The *R. stolonifer* parent, *Δcbs* strains and R*cbs* were grown on PDA plates. Colony morphology and conidiation were analysed after inoculating the PDA plates or screening plates that contained 2% CMC and 1% Congo red at 30 °C for 3 days. The spores of these three strains were inoculated on the fermentation medium in 5 L fermenters and cultured under the same conditions. By sampling at time interval of 6 h, the cell wet weight was recorded and the activity of cellulase was determined. The properly diluted supernatant (1 mL) were incubated with 1 mL substrate in sodium acetate buffer (0.1 M, pH4.8) at 50 °C for 30 min. Inactivated enzyme solution was used as a control group. The reduced sugars were measured according to the DNS method^36^. Filter paper (Whatman), CMC-Na and salicin were used as substrate to determine the activity of filter paper enzyme (FPase), endoglucanase (CMCase) and β-glucosidase, respectively^37^. Furthermore, the activity of cellobiohydrolase was determined with 0.5% (w/v) microcrystalline cellulose (Avicel, Sigma) at 50°C for 60 min. A unit of enzyme activity was defined as the amount of enzyme releasing one μmol of glucose equivalents per minute. Fermentation medium: 10% starch hydrolyzate/2% rice straw/2% glucose as single carbon source, 5% bran steep liquor (50 g wheat bran, boiled for 30 min, filtrated by 4 layer gauze, bring volume to 1000 mL with water), 0.5% NH_4_Cl, 0.5% KH_2_PO_4_, 0.4% MgSO_4_.7H_2_O, 0.4% CaCl_2_, 0.025% PEG4000, trace element solution, Tween-80, 1.2% methionine, 0.6% yeast extract.

### RT-qPCR analysis

Total RNA was extracted using Trizol (TAKARA, Dalian, China) and reverse transcribed using M-MLV reverse transcriptase (TAKARA, Dalian, China) following the manufacturer protocol. Quantitative RT-qPCR was conducted using SYBR Green Mix according to the manufacturer protocol. The glyceraldehyde-3-phosphate dehydrogenase A gene (*gpdA*) was used as the reference gene. RT-qPCR analysis was performed using the iQ5 Multicolor Real-Time PCR Detection System (Bio-Rad). All primers used in the study were presented in the Table S3.

### Statistics

All *in vitro* experiments were performed in biological triplicate. Statistical significance of the difference among groups was calculated using Student’s *t*-test. Data are presented as means ± s.e.s. P < 0.05 was considered to represent a statistically significant difference.

## Electronic supplementary material

Supplementary information
