# Supplementary material for: Self-induction system for cellulase production by cellobiose produced from glucose in Rhizopus stolonifer
Source: Sci Rep. 2017 Aug 31;7:10161. doi: 10.1038/s41598-017-10964-0 (PMC5579273; doi:10.1038/s41598-017-10964-0)
Supplement: Supplementary file 1 — Supplementary information [file 41598_2017_10964_MOESM1_ESM.pdf]

Supplementary information

Self-induction system for cellulase production by cellobiose produced from glucose in *Rhizopus stolonifer*

Yingying Zhang[1], Bin Tang[1,2*] & Guocheng Du[1,3]

[1]School of Biotechnology, Jiangnan University, Wuxi 214122, China. [2]College of Biochemical Engineering, Anhui Polytechnic University, Wuhu 241000, China. [3]Key Laboratory of Industrial Biotechnology, Ministry of Education, Jiangnan University, Wuxi 214122, China. Correspondence and requests for materials should be addressed to B.T. (tangbin@ahpu.edu.cn).

Figure Legends

Supplementary Figure 1 Analysis of the intracellular carbohydrate components of *R. stolonifer* TP-02 cultivated in glucose medium. (a) Chromatographic analysis of HPLC. (b) Liquid chromatography mass spectrometry (LCMS) analysis of the chemical cellobiose standard. (c) LCMS analysis of the disaccharide peak marked by box in a. (d) The time-course data of cellulose accumulation and cellulase induction of TP-02 cultured on glucose.

Supplementary Figure 2 Comparative analysis of the cellulase induction by glucose and cellobiose. Xylose was selected as the negative control (no cellulase activity was detected). The concentration of carbon source is 2%.

Supplementary Figure 3 Multiple alignments of CBS and its similar sequences from *Aspergillus niger* (GenBank No. CAK38334, and XP_001390453) and *Aspergillus oryzae* (GenBank No. EIT76265). The conserved catalytic center was underlined and marked by yellow. The characteristic sequences of BglB were marked by blue.

Supplementary Figure 4 Chromatographic analysis of the fermentation extracts of *E. coli* BL21 (pET28a-*cbs*) and the components of CBS mixture. (a) Standard sample: 1% (m/V) glucose and 1% cellobiose. (b) Blank Control: *E. coli* BL21 (pET28a). (c) Supernatant of the cell lysate from *E. coli* BL21 (pET28a-*cbs*). (d) Negative Control: *E. coli* BL21 (pET28a-*cbs*) without induced. (e-g): mixture of CBS (100 μg), UDPG (20 mg), and 0.5 mM ATP reacted at 30 ℃ for 30 min (e), 1 h (f), and 1 day (g). (h) Negative control of CBS mixture without ATP.

Supplementary Figure 5 Affinity analysis of CBS acted with UDPG (a), glucose (b) and cellobiose (c).The concentration gradient of UDPG, glucose, cellobiose and maltotriose (negative control) was 0 μM, 0.78 μM, 1.56

μM, 3.125 μM, 6.25 μM, 12.5 μM, and 25 μM. Specific information and data was shown in S4 Table.

Supplementary Figure 6 HPLC data of sugar components *in vivo* of *R. stolonifer Δugp* (a) and *Δcbs* (b) cultured on glucose medium. The FPA activities of parent TP-02, *Δcbs* and *Δugp* strains were determined (c). The supernatants of those three strains was analysed by SDS-PAGE (d).

Supplementary Figure 7 SDS-PAGE of supernatants of *R. stolonifer* parent, R*cbs* and *Δcbs* strains cultured on different medium.

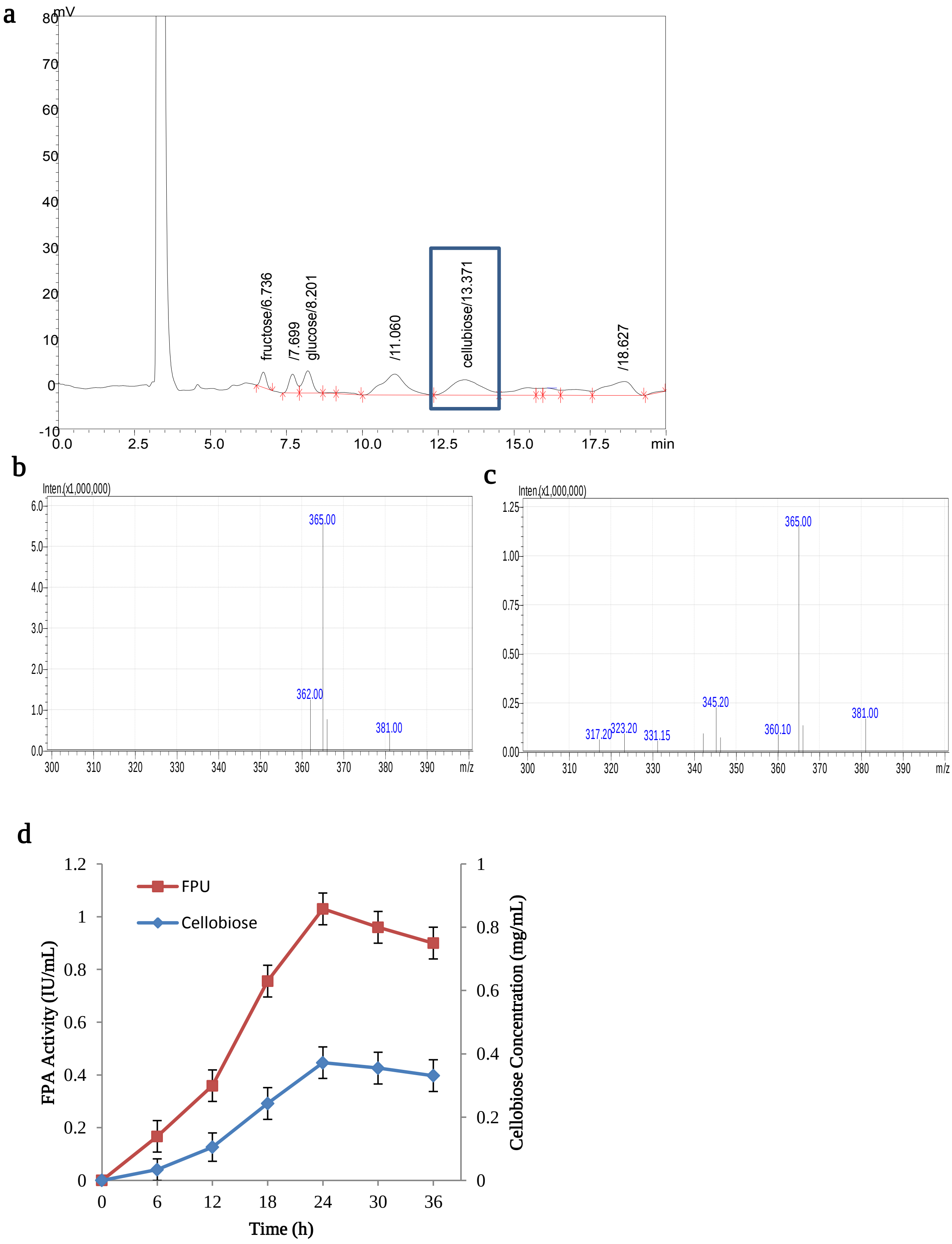

Supplementary Fig. 1

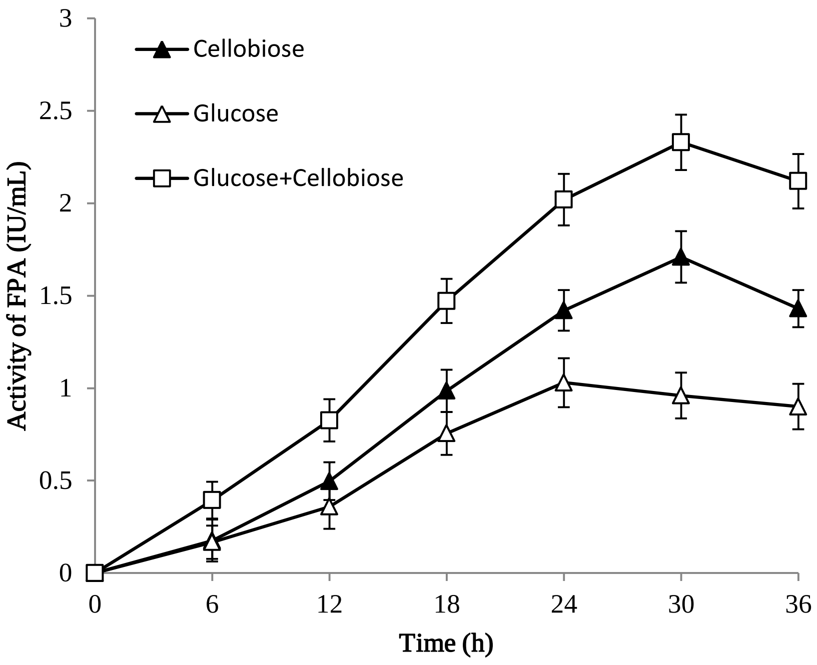

Supplementary Fig. 2

```
CBS             ------MDLQPLSRPSPSSVFLCCATKLASLAYLCTRTTGSTRTLPCSSNGMSIPPCTAV  54
EIT76265        MIS----------------EYVGEIEELPPDPRIWSGKRYRKYLYHTAQAAIWTSTVYYP  44
CAK38334        ------MDLQPLSRPSPSSVFLCCATKLASLANSVGRDRSRQYVYTTVNC----------  44
XP_001390453    MLSKLLYDIQELP----------------PDHRVVAGQRHRRLLYYVAHLASWTSNLYLV  44

CBS             FRHAGSLSSKRGRA----SYYVFSHYGVMISFSPCSVVRHQNHHHSKSQPRKR--LLHGT  108
EIT76265        IRLLMILLEAQQSWPMWLMLAVEAIFGRLSYQ--DQRLTVAAGGEPERGPRKRLRLRGSH  102
CAK38334        -----RVSTRRLPFRAN-EDAQANMFSVIWRH--DQLLTMLGGSASESQPRKRLRLHGTD   96
XP_001390453    LRLALCFSAVQQNWRAWLTLLVETVLAIIWRH--DQLLTMLGGSASESQPRKRLRLHGTD  102
                      .   :               . :      . :        :  ****  *

CBS             DLPQVDILVPCCGEPVDVILDTVRAACTMDYPVSSFRVRVLDDGASTELETAVAALRTEW  168
EIT76265        NLPRVDVLIPCCGEPVSVILDTVRAACTMDYPESQLRVLVLDDGASTQLRDAVSELHSKW  162
CAK38334        DLPQVDILVPCCGEPVDVILDTVRAACTMDYPVSSFRVRVLDDGASTELESAVAALRTEW  156
XP_001390453    DLPQVDILVPCCGEPVDVILDTVRAACTMDYPVSSFRVRVLDDGASTELESAVAALRTEW  162
                :**:**:*:*******.*************** *.:** ********:*. **: *:::*

CBS             PHLFYHTRGRQSGKVFAKAGIMNYALFTLQEKAPPAFCAIFDADSIPMPHFLRPTLPHLL  228
EIT76265        PYLFYHTRGRQSGRVFAKAGNLNYALFTVQKDTPPEFCAILDADSIPKPDFLRATLPHLL  222
CAK38334        PHLFYHTRGRQSGKVFAKAGNMNYALFTLQEKAPPEFCAIFDADSIPMPHFLRATLPHLL  216
XP_001390453    PHLFYHTRGRQSGKVFAKAGNMNYALFTLQEKAPPEFCAIFDADSIPMPHFLRATLPHLL  222
                *:***********:****** :******:*:.:** ****:****** *.*** ******

CBS             QTPEAVLLTTRQYFYNLPSGDPLSQSRLHIYTCENAELDRRGLAQDAGSGALFRRQAIID  288
EIT76265        LSPQTALVTTRQYFDNLPAGDPLSQSRLHFYTCQNAELDRCGRAIDAGSGAVFRRNAIID  282
CAK38334        QTPEAVLLTTRQYFYNLPSGDPLSQSRLHFYTCENAELDRRGLAQDAGSGALFRRQAIID  276
XP_001390453    QTPEAVLLTTRQYFYNLPSGDPLSQSRLHFYTCENAELDRRGLAQDAGSGALFRRQAIID  282
                 :*::.*:****** ***:**********:***:****** * * ******:***:****

CBS             SGGYPTYSFSEDWQLSLVLKGLGHRTIQVQQPLQFGLVPTSLDGHIAQRNRWHIGHSQQL  348
EIT76265        VGGYPTFSFSEDWQLSLILRGMGYRTVQVQEPLQFGLVPTSLEGHIAQRNRWHIGHSQQL  342
CAK38334        AGGYPTYSFSEDWQLSLVLKGLGHRTIQVQEPLQFGLVPTSLDGHIAQRNRWHIGHSQQL  336
XP_001390453    AGGYPTYSFSEDWQLSLVLKGLGHRTIQVQEPLQFGLVPTSLDGHIAQRNRWHIGHSQQL  342
                 *****:**********:*:*:*:**:***:***********:*****************

CBS             SVCSC-----RPQTRHFPARCSGVLHRMAS---------SSWLEAL-------------  380
EIT76265        FTLRPPTSSSIPRHLQWSIACGGLAITLGLVGHVIGYGAVPWLLMSRSLIPASSSFLIKT  402
CAK38334        WVLMPPTNKTLPRSLQWSIAQNGVFIMAGGLGCLISFAMVPLLLASGQLVPDISPLLAKA  396
XP_001390453    WVLMPPTNKTLPRSLQWSIAQNGVFIMAGGLGCLISFAMVPLLLASGQLVPDISPLLAKA  402
                 .         *:  ::    .*:    .             *

CBS             A-----VSFHLQWFHCFWHRAHSHRRQPGDPAAEQGGRVDRPRDPVGVAASVCHRVPEAA  435
EIT76265        QVILGLLHVSTMWAYGWLQTAHAGIR--GSPFSQLE--------NSWLAGAHLSAVIRFH  452
CAK38334        QFLLAVLHIALTWAYGLLQSAHTGFE--SFPFSRFE--------NIWLAGAHMHAVARFH  446
XP_001390453    QFLLAVLHIALTWAYGLLQSAHTGFE--SFPFSRFE--------NIWLAGAHMHAVARFH  452
                      : .   * :   : **:  .  . * :.             :*.:    * .
```

```
CBS             EVAECPLR-AAEDLRDGEWHEPEGGDNLPVEQLLQDEFRVQ---------------YFRD   479
EIT76265        FISSAPKGSFVTGSKENSWNRITKSSF--YKMLYQDLWQNGILQSICLLLATIAAMLLST   510
CAK38334        LLASRPKGSFVTGSSANSWNRSATPTS--FQKLYHNLWHNGIAYSIVVLLATIASIVYSI   504
XP_001390453    LLASRPKGSFVTGSSANSWNRSATPTS--FQKLYHNLWHNGIAYSIVVLLATIASIVYSI   510
                 ::. *    . .   ..*:.         : * :: ::
CBS             YIAAMADAYTLDGVNVRAYMAWSLME----------------------------------   505
EIT76265        WTTLTTTDSELLTTRLLTTIAWPPLLHICYLTVTNHWVPVAYLLSPPVYPARASQMAVLG   570
CAK38334        YTAITTTDDRLL-SRLLTTIAWPPMLHICYLSIVSYWAPVSYLLNPPRYPERKAGLALSE   563
XP_001390453    YTAITTTDDRLL-SRLLTTIAWPPMLHICYLSIVSYWAPVSYLLNPPRYPERKAGLALSE   569
                : :  :    *   .: : :**  :
CBS             ---------------------------------------------
EIT76265        SKE------------------------------------------   573
CAK38334        TGVVLPSPEVQQAAMVLGKAPVGFYRQCVVVVLVLGGLLLGGLTL   608
XP_001390453    TGVVLPSPEVQQAAMVLGKAPVGFYRQCVVVVLVLGGLLLGGLTL   614
```

Supplementary Fig. 3

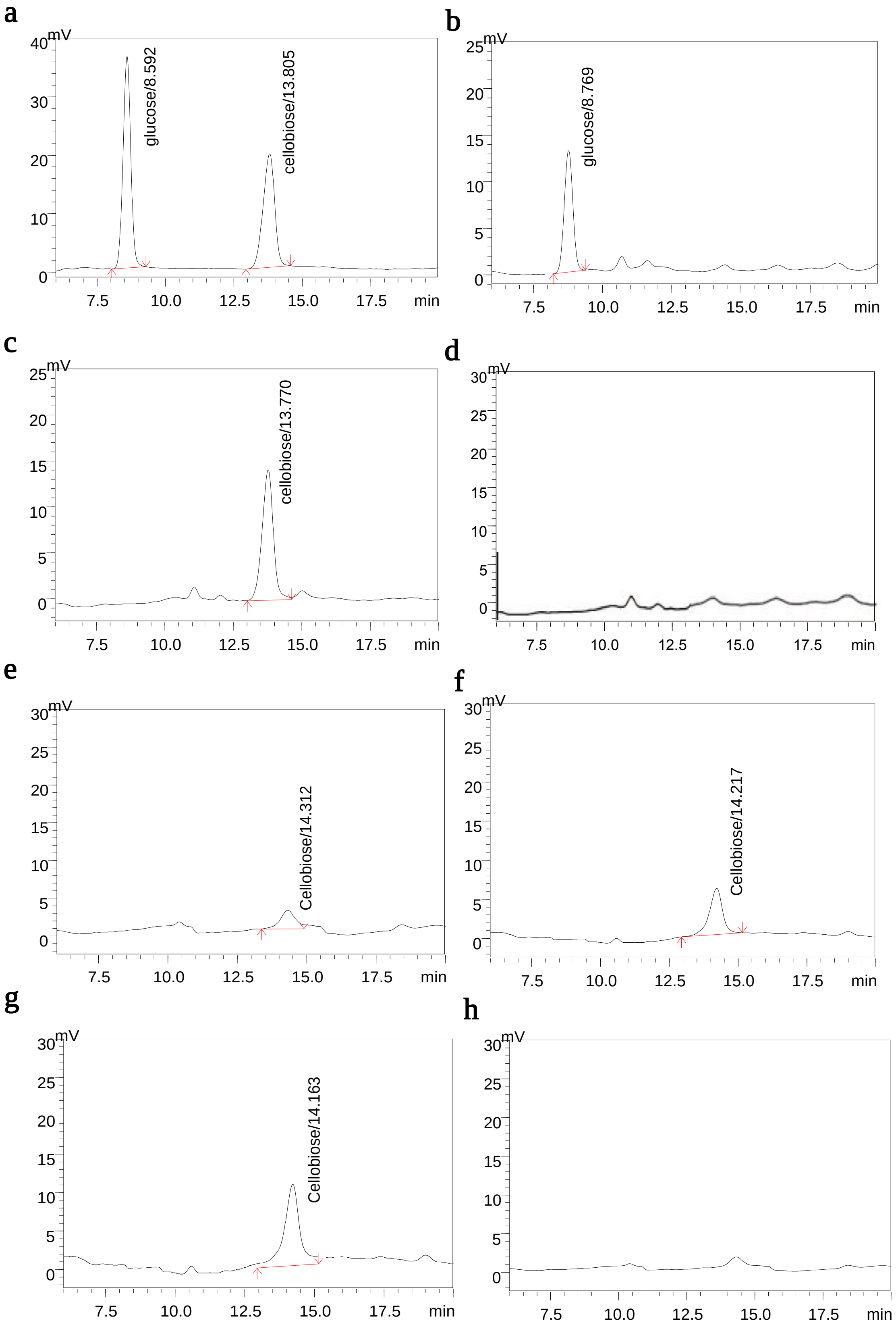

Supplementary Fig. 4

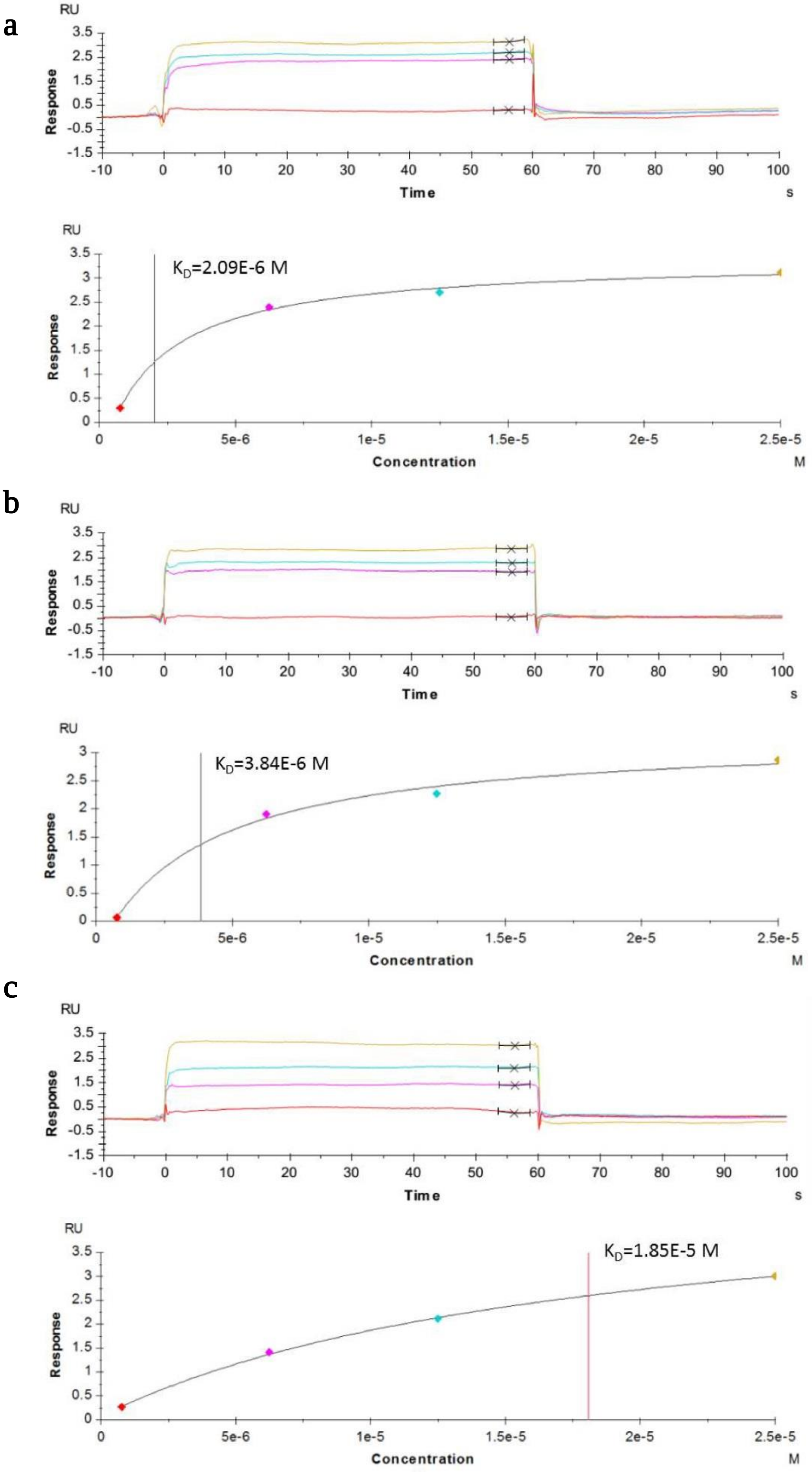

Supplementary Fig. 5

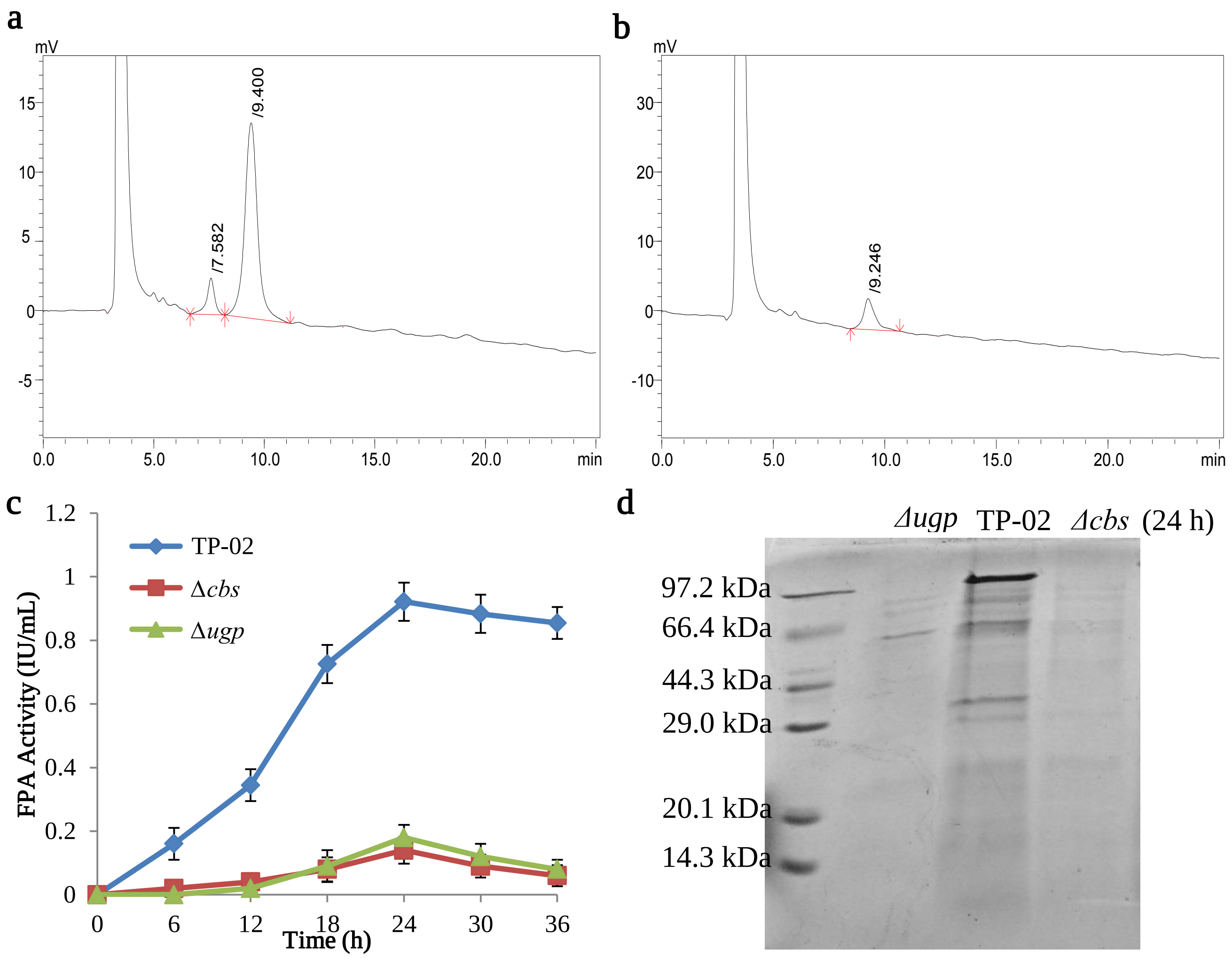

Supplementary Fig. 6

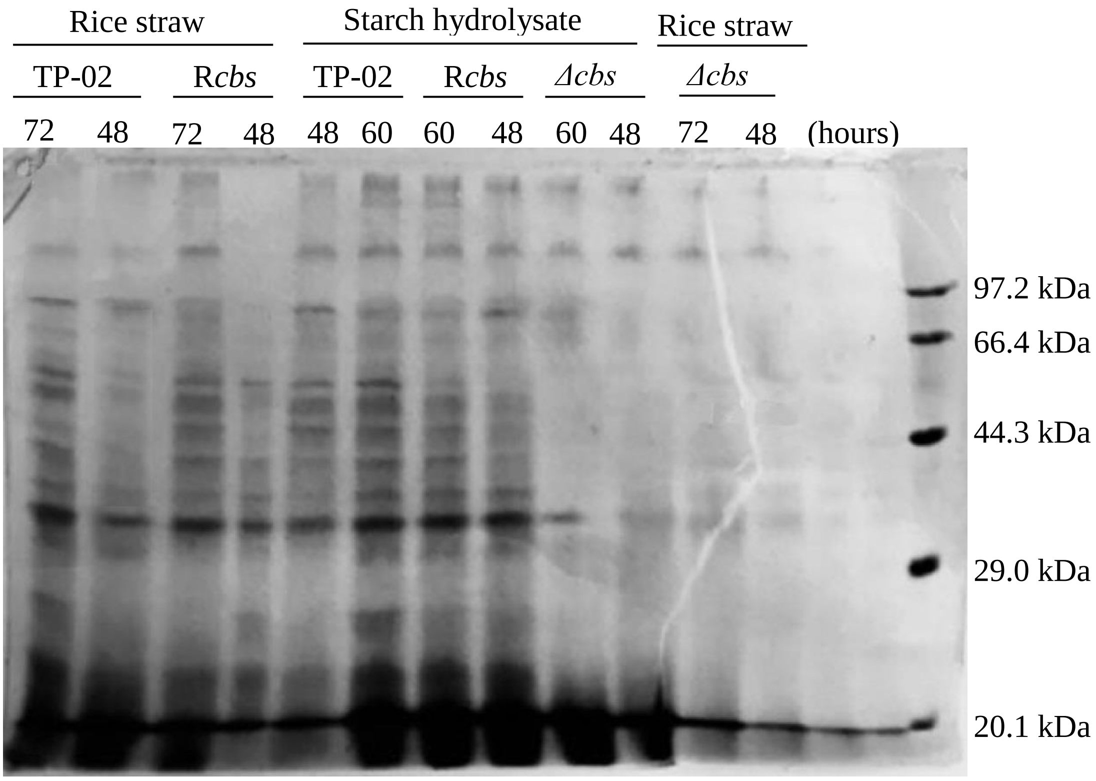

Supplementary Fig. 7

Tables

Supplementary Table 1 The sequences of primers for TAIL-PCR.

Products	Primers	Sequences(5'-3')
Upstream flanking sequence	USP1	TGATACCAGCCTTGGCGAAGAC
	USP2	GCTCTGTGGAAGCACCATCGTC
	USP3	ATGACGTCGACAGGCTCTCCG
	LAD1	BNBNNNGGTT
Downstream flanking sequence	DSP1	GTCACGCCTCCACATCTACACC
	DSP2	TGGCAGCTGTCGCTTGTTCTG
	DSP3	ACCGGTGGCACATTGGCCAC
	LAD2	BDNBNNNCGGT

Supplementary Table 2 The sequences of primers for Overlapping-PCR.

Products	Primers	Sequences(5’-3’)
	ugp-F	ATGTCCTCCGAGATGGCAACCC
	*ugp*Hyg-F1	GGTCCCAAGTCGGTCATCGAGGTCATGAAAAAGCCTGAACTCACCGCG
⊿ugp	*ugp*Hyg-F2	CGCGGTGAGTTCAGGCTTTTTCATGACCTCGATGACCGACTTGGGACC
(*hyg-ugp*)	*ugp*Hyg-R1	ACTCGTCCGAGGGCAAAGGAATAGCGTGACGGCATGTCTTTCCTCGAC
	*ugp*Hyg-R2	GTCGAGGAAAGACATGCCGTCACGCTATTCCTTTGCCCTCGGACGAGT
	ugp-R	CTAGTGCTGGAGAAGTCTCTAGC
	cbs-F	ATGGACCTCCAACCTTTATCTCG
	*cbs*Hyg-F1	GTCGAGGTCTTGCTCAGGATGCAGATGAAAAAGCCTGAACTCACCGCG
⊿cbs	*cbs*Hyg-F2	CGCGGTGAGTTCAGGCTTTTTCATCTGCATCCTGAGCAAGACCTCGAC
(*hyg-cbs*)	*cbs*Hyg-R1	ACTCGTCCGAGGGCAAAGGAATAGGCTCCGGGGCCCTCTTCCGGAGAC
	*cbs*Hyg-R2	GTCTCCGGAAGAGGGCCCCGGAGCCTATTCCTTTGCCCTCGGACGAGT
	cbs-R	TTACTCCATCAAACTCCACGCC
	clr1-F	ATGAGCAAGAGAGACACCCAAG
	*clr1*Hyg-F1	ACTGGACCCCTCAGTGCGTGACGATGAAAAAGCCTGAACTCACCGCG
⊿clr1	*clr1*Hyg-F2	CGCGGTGAGTTCAGGCTTTTTCATCGTCACGCACTGAGGGGTCCAGT
(*hyg-clr1*)	*clr1*Hyg-R1	ACTCGTCCGAGGGCAAAGGAATAGTGCACAAAGTGTCTGGAGGCTTC
	*clr1*Hyg-R2	GAAGCCTCCAGACACTTTGTGCACTATTCCTTTGCCCTCGGACGAGT
	clr1-R	CTACCCAATACTTTACTATTCTTC
	clr2-F	ATGCCTACGTATCGCATTCGCTGC
	*clr2*Hyg-F1	GTGGTTACGAATATGCGAGTCACATGAAAAAGCCTGAACTCACCGCG
⊿clr2	*clr2*Hyg-F2	CGCGGTGAGTTCAGGCTTTTTCATGTGACTCGCATATTCGTAACCAC
(*hyg-clr2*)	*clr2*Hyg-R1	ACTCGTCCGAGGGCAAAGGAATAGGACAGCAATACTTACACGAGTC
	*clr2*Hyg-R2	GACTCGTGTAAGTATTGCTGTCCTATTCCTTTGCCCTCGGACGAGT
	clr2-R	CTAAGCGTTCGCCGATCTCATCAC

Supplementary Table 3 The sequences of primers for RT-qPCR.

Gene	Primers	Sequences(5’-3’)
gpdA	GPDA-F	TACCGCTGCCCAGAACATC
	GPDA-R	GGAGTGGCTGTCACCGTTC
eg	EG2-F	TTATTGGGTTTGTTGTCAGGC
	EG2-R	GTGCTTTGAATTGATTGCTCC
bg	BG3-F	CGAGGACATTGCCTTGCTGA
	BG3-R	GTTTGTGGAGGGAATAGTGGG
cbh1	CBH1-F	CTTATTGTGGAGGCGGTTGC
	CBH1-R	CAGGTGGTATCGGTGGAGC
cbh2	CBH2-F	CCTGGCTATCCCATCCCTC
	CBH2-R	CGTTCTGGGCTTTGATGTCG
cbs	CBS-F	TGGACACATTGCACAGCGC
	CBS-R	TCGCCCGCCTTGTTCTGC
clr1	CLR1-F	GTCTACCTCTACCGCTGC
	CLR1-R	GTTGAGGAATCGCTTTAC
clr2	CLR2-F	TATCCGCTGCTGCCAGATC
	CLR2-R	GATGCTATAGTCGGTCAGTC

Supplementary Table 4 The binding parameters of the interaction between immobilized CBS and test compounds

Compounds	CAS No.	K_D(M)
Uridine diphosphate glucose (UDPG)	28053-08-9 (Anhydrous) / Sigma	2.09E-6
Glucose	50-99-7	3.84E-6
Cellobiose	528-50-7 / Sigma	1.85E-5
Maltotriose	113158-51-3 / Sigma	>1E-4
